# Innovation in Brain Tumor Treatment

**DOI:** 10.3390/brainsci16050475

**Published:** 2026-04-29

**Authors:** Terry Lichtor

**Affiliations:** Department of Neurosurgery, Rush University Medical Center Chicago, Chicago, IL 60612, USA; terry_lichtor@rush.edu

A dramatic increase in knowledge regarding the molecular biology of brain has been established over the past few years. Recent new avenues regarding the role of microRNAs, along with further understanding of the importance of angiogenesis, immunotherapy, and explanations underlying tumor resistance to radiation therapy have been developed. Improvements in surgical management, including improvements in imaging, as well as issues concerning tumor-induced epilepsy, have also been explored.

Antigenic differences between normal and malignant cells in cancer patients form the rationale for clinical immunotherapeutic strategies. While the central nervous system has traditionally been considered an immune-privileged site, several studies have demonstrated the potential efficacy of immunotherapy in the management of primary and secondary brain tumors. In addition, work has been conducted on the regulation of immune checkpoint inhibitors, which are capable of blocking molecules involved in inhibiting immune cells, thereby stimulating the T-cell response against various tumors, including brain tumors.

The goal of cancer therapy is the elimination of every remaining tumor cell from the patient. It is unlikely that a single form of therapy will be capable of achieving this goal. For this Special Issue, a review of novel and innovative treatments for these tumors is presented. It is hopeful that new strategies will be efficacious in the treatment of these tumors. Several important papers on the development of brain tumor treatments are included in this special edition.

Rafael Badalotti et al., in a research report titled “Gene Expression of GABAA Receptor Subunits and Association with Patient Survival in Glioma”, reported an overall association between higher expression of most genes encoding GABAA receptor subunits and better prognosis in different types of gliomas. In patients with high-grade gliomas, high expression of *GABRA2* was associated with shorter survival, whereas, in contrast, higher levels of *GABRB3* were associated with a better prognosis, indicated by longer survival. These findings support the possibility that down-regulation of GABAA receptors in glioma contributes to promoting tumor progression by reducing negative inhibition. These results might contribute to further evaluation of GABAA receptors as therapeutic targets in glioma.

Yunyang Zhu et al., in a research report titled “A Prediction Model for Deciphering Intratumoral Heterogeneity Derived from the Microglia/Macrophages of Glioma Using Non-Invasive Radio-Genomics”, demonstrated that risk stratification based on microglia/macrophages can effectively predict prognosis and immune functions in glioma. High-risk groups displayed an active microenvironment associated with microglia/macrophages. Furthermore, differences in somatic mutation rates, such as IDH1 missense variant and TP53 missense variant, were observed between high- and low-risk groups. Lastly, a radio-genomics model utilizing five features from magnetic resonance imaging (MRI) T2 fluid-attenuated inversion recovery (FLAIR) effectively predicted the risk groups.

Yu Wang et al., in a research report titled “Effect of Different Timing of Local Brain Radiotherapy on Survival of EGFR-Mutated NSCLC Patients with Limited Brain Metastases”, reviewed a study on radiotherapy and brain metastases. Although epidermal growth factor receptor (EGFR) and tyrosine kinase inhibitors (TKIs) have been the first-line therapy for EGFR-mutant lung adenocarcinoma (LAC) patients with brain metastases (BMs), the role and optimal timing of brain radiotherapy remain controversial. The authors aimed to investigate the role of upfront brain stereotactic radiotherapy (SRS) versus the impact of deferred radiotherapy on the clinical outcomes of these patients. Compared with upfront TKI therapy, the combination of upfront stereotactic radiosurgery with TKIs can improve intracranial progression-free survival and progression-free survival in patients with EGFR-mutant lung adenocarcinoma with synchronous brain metastases. The addition of upfront brain stereotactic radiotherapy was useful for the management of the original intracranial metastatic lesions.

Jiajun Zhou et al., in a research review titled “CNS Germ Cell Tumors: Molecular Advances, Significance in Risk Stratification and Future Directions”, reviewed central nervous system germ cell tumors, a subtype of intracranial malignant tumors. Current diagnostic methods in clinical practice have notable limitations, and treatment strategies struggle to achieve personalized therapy based on patient risk stratification. Advances in molecular genetics, biology, epigenetics, and understanding of the tumor microenvironment support the diagnostic potential of associated molecular alterations, aiding risk subgroup identification at diagnosis. Furthermore, the authors suggest that the application of novel therapeutic approaches targeting chromosomal alterations, mutated genes and altered signaling pathways, methylation changes, microRNAs, and immune checkpoints may be beneficial. For patients with refractory or recurrent CNS germ cell tumors who are resistant to conventional treatment methods, the potential benefits of new therapies targeting their molecular characteristics warrant further investigation. CNS germ cell tumors remain a relatively rare disease. It is important to investigate whether patients with unique molecular features can benefit from lower-intensity treatment approaches and reduce the long-term adverse effects typically associated with conventional treatments. Nevertheless, CNS germ cell tumors remain a relatively rare disease.

Terry Lichtor et al., in a research review titled “Cytokine Gene Vaccine Therapy for Treatment of a Brain Tumor”, explored the applications of cytokines such as IL-2 and IL-15 in the treatment of gliomas. Treatments with either a poxvirus genetically engineered to secrete IL-15 or allogeneic fibroblasts transfected with tumor DNA and engineered to secrete IL-2 were shown to be effective strategies for extending the survival of mice with malignant brain tumors following intracerebral injection of the treatment cells. DNA-based cytokine-secreting vaccines offer several unique advantages, supporting their further development as an immunotherapeutic treatment option for patients with a variety of tumors in general and for those with malignant intracerebral tumors. The use of viruses engineered to secrete cytokines remains another treatment option and requires further study regarding the management of these tumors.

Richard E Kast et al., in a research review titled “Non-Invasive Ultra-Low Intensity Light Photodynamic Treatment of Glioblastoma with Drug Augmentation: LoGlo PDT Regimen”, reviewed ultra-low intensity light photodynamic treatment of glioblastoma. Oral 5-aminolevulinic acid (5-ALA) is an FDA/EMA-approved drug that is selectively taken up by malignant cells, including glioblastoma cells. In photodynamic treatment of glioblastoma, intense intraoperative light causes glioblastoma tissue that has taken up 5-ALA to generate cytotoxic reactive oxygen species. The requirement for intense light flux has restricted photodynamic treatment to a single one-hour intraoperative session. The LoGlo photodynamic treatment approach uses both drug augmentation and prolonged, ultra-low, non-invasive transcranial light delivery sessions for repetitive, non-invasive 5-ALA photodynamic treatment of glioblastoma.

